# What do Key Stakeholders Think About HIV Self-Testing in Canada? Results from a Cross-Sectional Survey

**DOI:** 10.1007/s10461-017-1764-z

**Published:** 2017-04-24

**Authors:** N. Pant Pai, M. Smallwood, D. Gulati, N. Lapczak, A. Musten, C. Gaydos, C. Johnston, M. Steben, T. Wong, N. Engel, J. Kim

**Affiliations:** 1 Division of Clinical Epidemiology and Infectious Diseases, Faculty of Medicine, McGill University Health Centre, Montreal, QC Canada; 2 Maastricht University, Maastricht, The Netherlands; 3 REACH, Toronto, ON Canada; 4 Division of Infectious Diseases, Johns Hopkins University, Baltimore, MD USA; 5 Canadian AIDS Treatment Information Exchange, Toronto, ON Canada; 6 INSPQ, Montreal, QC Canada; 7 Public Health Agency of Canada, Toronto, ON Canada; 8 National Labs Canada, Winnipeg, MB Canada

**Keywords:** HIV, Self-testing, Survey, Mixed-methods, Stakeholders

## Abstract

Human immunodeficiency virus (HIV) self-testing presents an empowering alternative to facility-based testing for reaching undiagnosed HIV infected individuals, but is not currently available in Canada. We surveyed stakeholders (clinical providers, public health professionals, researchers) engaged in HIV testing initiatives nationwide to identify the concerns, opportunities and challenges to implementing HIV self-testing in Canada. An online cross-sectional survey was disseminated by the Canadian Institutes of Health Research Centre for REACH 2.0 National HIV & sexually transmitted and blood borne infections working group to stakeholders nationwide, with a target sample size of 200. Quantitative and qualitative data were analyzed using a mixed-methods, respondent-informed approach, to inform subsequent HIV self-testing in a country where self-testing is not yet accessible. A total of 183 responses were received. A majority (70.7%) (128/181) felt that self-testing was a necessary investment to reach the undiagnosed. 64.6% (117/181) felt that self-tests should be made available to their clients and 71.5% (128/179) of respondents agreed that self-test instructions required improvements. However, 50% (90/180) felt that self-testing will pose an economic challenge to current HIV testing models. Regardless, 21% urged for timely action and availability of HIV self-tests. Thematic analyses reflected the following concerns: (a) need for affordable self-tests, (b) need for expedited, customized, and accessible linkages to counselling, (c) concern for patients to cope with positive self-test results, (d) accuracy of self-tests to detect acute HIV and (e) liability in the context of non-disclosure. Stakeholders agreed to the provision of an option of HIV self-testing to reach the undiagnosed individuals. Concerns regarding costs and accuracy of self-tests, expedited linkages to counselling, and integration of self-test within prevailing HIV testing models, will need to be addressed before their widespread implementation.

## Introduction

Approximately 21% of the estimated 75,500 Canadians living with HIV in 2014 were undiagnosed [1]. Individuals with undiagnosed HIV may face barriers, such as stigma, social visibility, privacy, and long wait times in health facilities, limiting the uptake of conventional, facility-based HIV testing. HIV self-testing is a process that allows individuals to perform an initial HIV test and interpret results without having to engage in-person with the healthcare system [2, 3]. This presents an empowering alternative to conventional testing for key populations impacted by the HIV epidemic. The benefits of self-testing are privacy, confidentiality, convenience, empowerment, and knowledge of serostatus; [4–7] yet concerns regarding expedited linkages to care and counselling, impede its global operationalization [5, 8, 9].

The World Health Organization (WHO) has recently released new guidelines for HIV self-testing, recommending that self-testing be offered as an additional option for HIV testing [3, 5]. These guidelines will catalyze the uptake of self-testing initiatives. According to the WHO, if HIV self-testing is sensibly implemented with community involvement, it may present an opportunity to increase knowledge of HIV status while maintaining the five Cs of HIV testing and care: consent, confidentiality, counselling, correct results and linkage to care [5].

The oral HIV self-test (OraQuick) is available in the United States [10], and blood-based self-tests are available in the United Kingdom (BioSure HIV self-test) [11] and France (Autotest VIH) [12]. Self-tests have not yet been approved for use in Canada (by Health Canada), nor have manufacturers applied for approvals. Research data from Canada are sparse with only two studies conducted to date: one in student populations [13], and the other in men who have sex with men (MSM) [14]. At the Canadian Institutes of Health Research (CIHR) Centre for REACH in HIV/AIDS (REACH 2.0) National HIV & STBBI testing working group, we have noted this limited availability of HIV self-testing data which is needed to guide approvals and operationalization in Canada. As the world is opening up to the concept self-testing for HIV, Canada must keep up.

In this context, we set out to survey the knowledge, opinions, and perceptions of Canadian stakeholders of REACH 2.0 on the hypothetical implementation of HIV self-testing in Canada, through a mixed-methods approach.

## Methods

Between June and December 2015, with the support of REACH 2.0 and its partners (Canadian AIDS Treatment Information Exchange (CATIE), the CIHR Canadian HIV Trials Network (CTN), and the Canadian Aboriginal AIDS Network (CAAN)), we offered an online survey to all stakeholders within this network, across all provinces. The sampling frame consisted of all stakeholders involved in HIV self-testing initiatives across Canada. Our target sample size was 200. Participants were recruited via convenience sampling method to maximize the number of responses among this relatively specific group, and the survey was disseminated by email via various Canadian HIV organizations (e.g. CATIE, REACH 2.0, CAAN, CTN).

The survey was approved by the Institutional Review Board of McGill University, Montreal. It was offered in French and English to minimize selection bias and to maximize representation. Participants provided consent by submitting the completed questionnaire, being able to withdraw at any time.

The survey was created using SurveyGizmo™ [15] and contained 34 items. The survey instrument was designed based on past surveys conducted internationally on HIV self-testing [2, 8, 9], and was previously validated in South Africa. It was pre-piloted amongst stakeholders from the REACH 2.0 working group, and feedback was used to make improvements and adjustments to the design.

Survey questions aimed to collect respondents’ demographic information, practice setting, knowledge, attitudes, and perceptions of self-testing for HIV in Canada. Because self-testing is not yet available in Canada, the respondents of our survey were unlikely to have any first-hand experience with self-tests for HIV. Thus, questions were designed to be speculative/hypothetical, assessing perceptions and attitudes, rather than lived experiences. Topics such as the need for self-testing, its perceived barriers, the harms, risks, benefits, and perceived client preferences for HIV self testing were explored. The question format included a variety of questions, such as multiple-choice (17 questions), three-level Likert scale (10 questions), yes/no (5 questions), and open-ended (2 questions). Respondents were also given the opportunity to provide comments.

A mixed methods approach was used to analyze quantitative and qualitative data.

Quantitative data were summarized using frequencies and proportions with 95% confidence intervals (Stata IC/13, Stata Corp, College Station, TX, 2013). For key questions, proportions stratified by primary job function were grouped into frontline care providers (healthcare providers, frontline healthcare professionals, and healthcare managers) and all other stakeholders (researchers, policy makers, and other stakeholders). If proportions were found to differ by primary job function, further analysis was performed using a proportional odds logistic regression model [16]. The dependent variable was ordinal with three possible outcomes: disagree, neutral, or agree.

Qualitative data was acquired through the two open-ended questions, and comments for each question (23 total). Respondents were able to freely voice their views and concerns regarding supervised HIV self testing regardless of the content of the specific question. Data was organized using NVIVO software (NVivo qualitative data analysis Software; QSR International Pty Ltd. Version 11.1.1). An initial coding scheme was tested and further refined by the co-authors in the course of the analysis. It was based on examining patterns and linkages between the codes and developing themes [17], and was informed by extant literature [13].

## Results

**Fig. 1:**
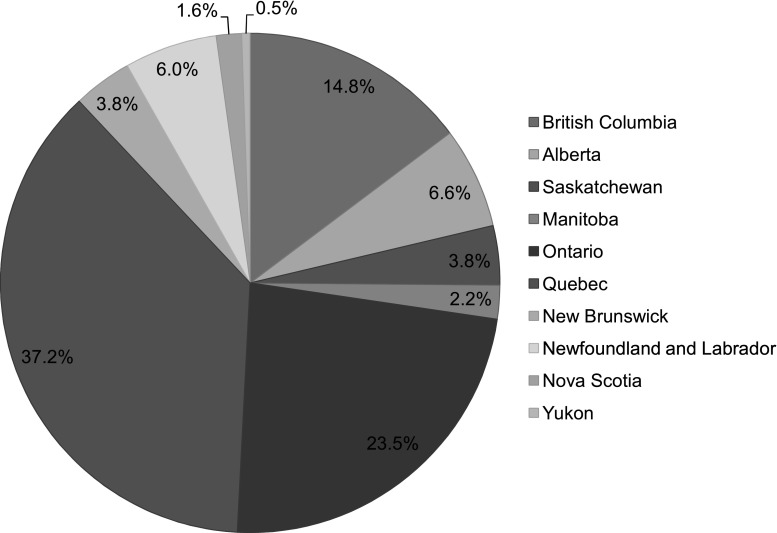
Province of employment of survey respondents, n = 183, Canada 2015

**Fig. 2:**
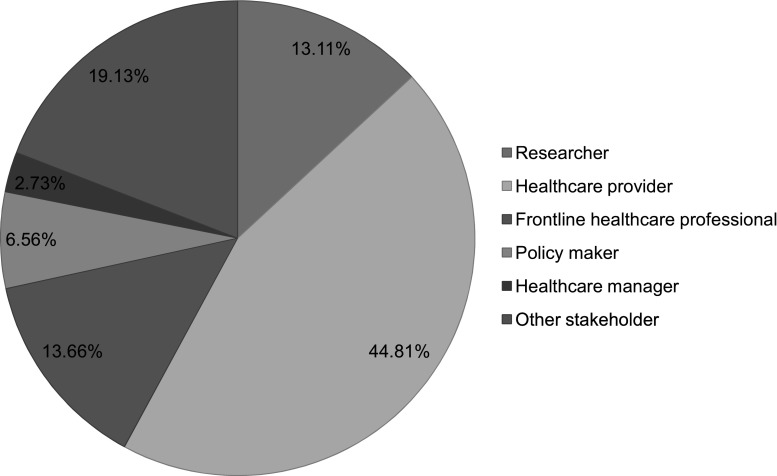
Primary job function of respondents, n = 183, Canada 2015

Table 1 summarizes descriptive statistics. We received a total of 183 responses to the survey, of which 72% were completed in English. Stakeholders from 10 different provinces and territories responded. Quebec and Ontario constituted the majority of stakeholders (37 and 24% respectively) (Fig. 1). Healthcare providers constituted 45% of stakeholders; followed by “other” stakeholders (19%), which included AIDS organizations, community outreach workers, and social workers, among others (Fig. 2).

**Table 1:** Descriptive characteristics of survey respondents

Question	Responses (n)	Response	(%)	95% CI
Survey language	183	English	72.1	65.1–78.2
		French	27.9	21.8–34.9
Years of work in HIV	180	0–5 years	30.6	24.2–37.7
		6–10 years	24.4	18.7–31.3
		11–15 years	10.0	6.4–15.4
		16–20 years	12.8	8.6–18.6
		21–25 years	7.8	4.6–12.8
		25+ years	14.4	10.0–20.4
Practice setting	111	Private clinic	10.8	6.2–18.2
		Community clinic	17.1	11.1–25.4
		Sexual health clinic	16.2	10.4–24.4
		Mobile clinic	1.8	0.4–7.1
		Hospital clinic	18.0	11.9–26.4
		University-based clinic	5.4	2.4–11.6
		Other setting	30.6	22.7–40.0
Type of testing offered^a^	109	Nominal (name-based)^b^	61.5	51.9–70.2
		Non-nominal (non-identifying)^c^	18.4	12.1–26.9
		Anonymous^d^	12.8	7.7–20.7
		Other	7.3	3.7–14.1
Percentage of testing that is POC	109	0%	26.6	19.1–35.8
		1–25%	32.1	23.9–41.6
		26–50%	12.8	7.7–20.7
		51–75%	3.7	1.4–9.5
		76–100%	15.6	9.9–23.8
		Not currently available	4.6	1.9–10.7
		Do not know	4.6	1.9–10.7
Population of clients you regularly test for HIV (% yes)^e^	112	MSM	70.5	61.3–78.3
		Bisexual or transgender	50.9	41.6–60.2
		Drug users	60.7	51.3–69.4
		Aboriginal	34.8	26.5–44.2
		Immigrants from HIV endemic countries	36.6	28.1–46.0
		Incarcerated	42.9	33.9–52.3
		Homeless or living in shelters	42.9	33.9–52.3
		Sex workers	50.0	40.7–59.3
		Other clients	27.7	20.1–36.8
Is rapid POC testing routinely used in your work setting?	108	Yes	47.2	37.9–56.8
		No	50.9	41.4–60.4
		Do not know	1.9	0.5–7.3

^a^Three options for testing are available in Canada, depending on the province: Nominal, non-nominal and anonymous (offered in 7 provinces)

^b^Nominal (name-based) testing: The health care provider ordering the test knows the identity of the person being tested, test is ordered using the name of the person being tested, and public health officials must be notified of a positive result

^c^Non-nominal (non-identifying) testing: Same as nominal testing, however the test is ordered using either a code or the initials of the person being tested

^d^Anonymous testing: Health care provider ordering the test does not know the identity of the person being tested, the HIV test is ordered using a code, test results not recorded on health care record of person being tested

^e^Proportions do not add up to 100% because respondents could select more than one response

Analyses by job function (all other stakeholders vs. frontline care providers) with ordinal logistic regression models (Table 2), demonstrated other stakeholders (n = 71) were more likely to respond in favour of self-testing than frontline care providers (n = 112). The proportional odds of selecting “agree” versus “neutral” or “disagree” (likewise, for selecting “agree” or “neutral” versus “disagree”) were 3.82 (95% CI 1.89–7.71, “HIV self-tests are an important approach to testing and should be made available to my clients”), 2.44 (95% CI 1.20–4.98, “investment in self-testing is necessary to help bring undiagnosed individuals into care”), and 2.64 (95% CI 1.39–5.02, “clients will benefit by having access to self-test kits”), comparing other stakeholders to frontline providers.

**Table 2:** Level of agreement on questions regarding self-testing, stratified by stakeholders (n = 71) vs. frontline care providers (n = 112) Proportional odds ratios (OR) are presented with their 95% confidence intervals (95% CI), comparing all other stakeholders to frontline care professionals

Survey question	HIV self-tests are an important approach to testing and should be made available to my clients				Investment in HIV self-testing is necessary to help bring undiagnosed individuals into care				In your opinion, your clients will benefit by having access to self-test kits			
	Agree	Neutral	Disagree	Proportional OR (95% CI)	Agree	Neutral	Disagree	Proportional OR (95% CI)	Agree	Neutral	Disagree	Proportional OR (95% CI)
Other stakeholders^*^	81.1% (56/69)	15.9% (11/69)	2.9% (2/69)	3.82 (1.89–7.71)	81.4% (57/70)	12.9% (9/70)	5.7% (4/70)	2.44 (1.20–4.98)	73.1% (49/67)	19.4% (13/67)	7.5% (5/67)	2.64 (1.39–5.02)
Frontline care professionals	54.5% (61/112)	26.8% (30/112)	18.8% (21/112)		64.0% (71/111)	24.3% (27/111)	11.7% (13/111)		50.9% (57/112)	31.3% (35/112)	17.9% (20/112)	

* Researchers, policy makers, and “other stakeholders” (e.g. community AIDS workers) were grouped together in this analysis

The majority of the qualitative data was analyzed from the responses to the comments provided alongside the non-open ended questions (23 questions). For the open-ended questions, 42% (77/183) of stakeholders provided responses to the first question (“Do you have any thoughts on what type of research is needed for HIV self-testing in Canada?”) and 40% (73/183) responded to the second question (“Do you have any thoughts on what is needed to approve HIV self-tests for Canadians?”).

Stakeholders generally responded in favour of self-testing, with 64.6% (95% CI 57.3–71.3%, n = 181) agreeing that HIV self-tests are an important approach to testing and should be made available to their clients. 70.7% (95% CI 63.6–76.9%, n = 181) agreed that investment in self-testing will be necessary to help bring undiagnosed individuals into care, and 59.2% of respondents (95% CI 51.8–66.2%, n = 179) felt that clients would benefit by having access to self-testing kits. Stakeholders expressed concerns regarding linking patients to clinical care and counselling. Of respondents, 71.5% (95% CI 64.4–77.7%, n = 179) agreed that before self-test kits are made widely available, self-test instructions must be improved to ensure users understand the need to access clinical care (e.g. confirmatory testing, counselling and treatment).

The following major themes emerged from the qualitative analysis. Where available, these were matched with corresponding quantitative findings.

### The Client’s Emotional and Cognitive Characteristics in Handling Test Results: “Fit to Test?”

Respondents highlighted several factors, such as a sufficient level of the client’s understanding of self-testing technologies, HIV and its prognosis, and integrity of the client’s mental health status and coping mechanisms as determinants for a client’s capability to handle a positive self-test result without psychological harm or endangering others, and to engage with counselling and care. We summarized these factors as “fitness to test”. This fitness to test also assumes literacy and understanding of the pros and cons of the testing process and entails proactivity on the part of the self-tester. Underlying many comments seemed to be an analysis of the respondent’s own client population, and in particular their psychological state and coping skills (e.g., insufficient trust and aversion to pre-test counselling and interrogation about one’s sex life) which may deter clients from conventional HIV testing. HIV self-testing may allow clients who experience these feelings/perceptions to access HIV testing anyway, offering an advantage. Others emphasized that the act of self-testing itself can be an indicator for inward (seeking counselling and care) and outward (preventing transmission to future partners) responsibility. One health care provider noted: “The majority of my clients would be able to understand the accuracy of the technology, the implications of having a positive test result, and would have the coping skills to actively seek out the services available (…)” when asked about the importance of self testing and whether it should be made available to clients. For clients considered fit to test by the stakeholder, self-testing can be empowering, by allowing clients to autonomously diagnose their infection and seek treatment. In contrast, in a traditional test setting, this is (partially) outsourced, and responsibility is passed from the client to the healthcare provider, who makes the decision to test.

When asked if self-tests are too difficult for clients to interpret, 50% (95% CI 42.7–57.3%, n = 180) of respondents disagreed, 21.1% (95% CI 15.7–27.8%) of respondents were neutral, and 14.4% (95% CI 10.0–20.4%) agreed. Less than one-third of respondents (28.3%, 95% CI 22.2–35.4%, n = 180) felt that self-tests put too much responsibility on clients (42.2% disagreed, 95% CI 35.1–49.6%; 25% neutral, 95% CI 19.2–31.9%).

### The Next Steps: Linkage to Counselling and Care

Ensuring customized counselling and care, for both positive and negative results, was a primary concern of respondents as exemplarily noted by an activist and former physician when asked the type of research which was needed for HIV self-testing in Canada: “I think the link from self-test to counselling is the most important thing!”.

The specifications and suitability of the services offered to clients were perceived to depend on pre-existing knowledge of HIV and self-testing, educational background, cognitive ability, and reading and language skills. Here the difference between accessibility and acceptability was highlighted, as a tester may not consider a service that is existing and accessible acceptable or appropriate for their needs. Stakeholders expressed in their comments that their approval of self-testing depends upon their perception of how likely an HIV self-test will lead to a confirmed diagnosis and linkage to care, as highlighted by a health care manager when asked about the relevance of self-testing and whether to make it available to clients: “This is contingent on patients with positive results having the ability and the means to seek appropriate care.” This comment also highlights the relevance of “fitness to test” for successful linkage to counseling and care. Respondents indicated that improvements to self-test instructions were needed to ensure clients access clinical care, and 70.6% (95% CI 63.4–76.9%, n = 177) of respondents considered phone-based counselling and linkage to care would be a good option while offering supervised or unsupervised self-testing. In addition, 60.1% (95% CI 52.7–67.1%, n = 178) of respondents agreed that clients who self-test false-negatively will be less likely to engage with HIV care. However, respondents generally disagreed that clients self-testing positive will be less likely to engage with HIV care (only 11.7%, 95% CI 7.7–17.3% n = 180, of respondents agreed). One quarter (25%; 95% CI 19.2–31.9, n = 180) of respondents felt that self-tests have the potential to do more harm than good.

### Self-Test Characteristics: Quality and Accuracy

Of respondents, 34.4% (95% CI 27.8–41.8%) admitted a deficit in knowledge about self-test’s sensitivity, highlighting a need for awareness and education among stakeholders if self-tests are to be approved and made available in Canada. Quality (accuracy) of self-testing was perceived to be determined by sensitivity and specificity, and the ability to detect HIV during the window period. Respondents didn’t want quality of testing (and counselling) to be compromised for increased testing numbers.

### Testing Environment, Test Administration and “Subjective Safety”

**Fig. 3:**
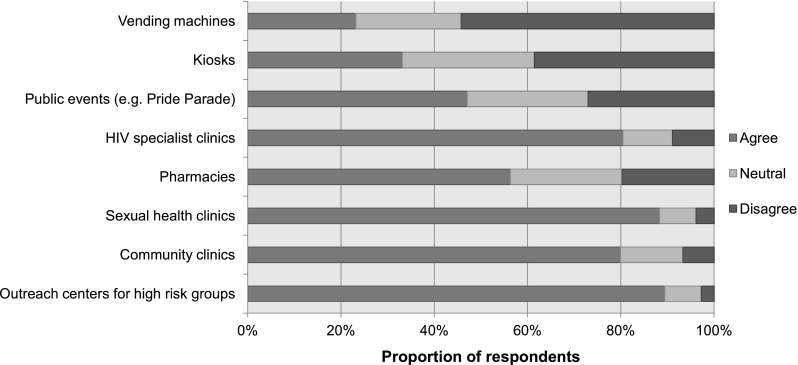
Responses to the question, “Which of the following would be suitable places for HIV self-tests to be made available to the public?”, n = 164, Canada 2015

Self-tests can be made available in a variety of settings, for example home, office, private spaces in clinics, outreach settings, and pride parades (Fig. 3). Several respondents perceived safety, privacy and confidentiality as key determinants of the quality of the self-testing environment, and a distinction was made between the self-testing environments’ ability to provide these determinants, as compared to traditional test settings. As an example for several responses in the same line, a medical director of a clinical microbiology laboratory noted: “(…) I think this type of testing provides clients with the opportunity to test anonymously in the safety and comfort of their home (…)”. Depending on the location where self-tests are made available (e.g. gay pride parade) respondents could not assume privacy, safety, and confidentiality would be ensured, as highlighted by a stakeholder within an AIDS Service Organization when asked about suitable places for self-testing in this statement: “If available at public venues such as Pride Parade, Pharmacies or Kiosks, steps should be taken to ensure set up is respectful of person’s privacy and confidentiality.” Traditional settings (e.g. clinics, health centres) were understood to provide better quality. According to several respondents, privacy and anonymity are particularly relevant for high risk or vulnerable populations, and for this reason, self-testing was perceived to provide a safe testing experience, reaching more individuals in remote areas. Through qualitative responses, the concept of “subjective safety” was developed: the array of individual factors that ensure a comfortable testing experience.

Privacy concerns were mentioned in relation to technological innovations, such as smartphone applications. 53.7% (95% CI 46.2–61.0%, n = 177) of respondents agreed that clients are concerned about protection of health information and confidentiality with online testing. 42.4% (95% CI 35.2–49.8, n = 177) of respondents felt that clients would be comfortable with using mobile phones to engage with counselling, however 24.9%, (95% CI 19.0–31.8%, n = 177) were unsure whether clients would be comfortable doing so. Many stakeholders (21%) expressed a desire for prompt availability of self-tests in Canada.

### Financial Aspects

Approximately 50% of respondents favoured either free or $0–5 CAD cost if self-testing had to be paid for by the individual. The inverse correlation between accessibility (esp. by marginalised groups) and (higher) cost was widely recognised, as noted by a policy maker when asked about the desired pricing of self-testing:“The higher the price, the more likely that marginalized individuals who are hard to reach will not access it”.

Fifty percent (95% CI 42.7–57.3%, n = 180) of respondents felt that self-testing (as an alternative strategy) poses a challenge to current business models for HIV testing (conventional and anonymous HIV testing and counselling); qualitative responses considered the impact that self-testing might have on general HIV testing budgets and related consequences for the overall availability of testing, should existing budgets not be expanded, as illustrated in this statement by a frontline outreach/education worker in the context of self-testing strategies in Canada: “The province of Ontario is already talking about taking away POC testing from underperforming clinics. If the province puts funding into self-testing, I would imagine that money will come from current testing budgets”. Some respondents expressed concerns about the potential for self-testing to economize HIV testing, as stated by a health care provider in the context of supervised self-testing in settings with high case loads: “We need to be careful not to address issues in resource allocation through compromising quality of care”. Moreover, the costs of self-tests were appraised as a determinant for specific groups to access self-testing, and respondents mentioned the need to consider the costs of pre- and post-test counselling.

## In Addition to These Themes Several Cross-Cutting Issues were Identified

### Stigma

Stigma appeared in different contexts as a recurring theme in the respondents’ comments. Several respondents illustrated it as a pervasive phenomenon and an emotional stressor to the client being tested, affecting the interaction between the client and healthcare provider and the likelihood of engagement and linkage across the HIV care continuum following testing. One health care provider noted “But there is a high level of stigma attached to this infection and this does not allow for a level playing field” when asked whether her or his clients would benefit by having access to self-testing. Self-testing was credited with the potential to reduce perceptions of stigma, as noted by a policy maker when asked where to make self-testing available: “The more access people have not only leads to de-stigmatizing, it also offers easy access”. Self-testing was also understood to counter HIV exceptionalism, as highlighted by a health care provider in relation to the most important benefit associated with self-testing: “I actually think that making self-testing something natural could reduce stigma (…)”.

### Liability

A few respondents noted the relevance of legal considerations of self-testing with respect to Canadian legislation, which requires individuals to disclose their HIV status before having sex that poses a “realistic possibility of HIV transmission” [18]. Some respondents also considered the implications for liability in a case of non-disclosure when a person was diagnosed through a self-test and did not receive HIV counselling and care. The usefulness of unsupervised self-testing in situations involving “illegal activity” leading to HIV exposure was highlighted by respondents, whereby testers can avoid disclosing such information to healthcare providers. This was summarized by a healthcare manager in the context of the use of self-tests in Canada with respect to their clients and patient populations: “Even persons who want to get life insurance/health insurance may want to do a self test before applying for insurance. They will not want to share their positive test result for the same reason/worry about being declined for insurance. There are also those who worry about being prosecuted for engaging in illegal behaviors, etc”.

## Discussion

This first Canadian survey highlighted many aspects with respect to the implementation of HIV self-testing in Canada. While many of these results are in agreement with extant literature on HIV self-testing, some are particularly relevant in the Canadian context [19]. A clear need for contextualized and tailored strategies for key populations emerged for Canadians, especially in light of two specific limitations: liability issues in cases of HIV non-disclosure, and self-testing being viewed as a challenge to the existing business models of HIV testing in various provinces. This survey also highlighted the potential of self-testing for vulnerable populations (e.g. MSM) in light of distrust in the healthcare system or (perceived) incompetence of its actors to provide appropriate and acceptable services (e.g. potential discomfort with revealing sensitive information such as sexual history to healthcare providers). Our survey findings concurred with an HIV stakeholders’ survey in South Africa [20], and a study which reviewed public readiness for self-testing in Kenya [21].

Operationalizing linkage to counselling and care was perceived by stakeholders to be the greatest challenge to self-testing, and pivotal for stakeholders’ approval of HIV self-testing in Canada. Phone-based linkages and smartphone applications were deemed acceptable if confidentiality of information was safeguarded, especially in light of liability issues in cases of HIV non-disclosure. This concern can be partially addressed with online or smartphone-based applications that conform to the Health Insurance Portability and Accountability Act of 1996 (HIPAA) standards [22]. Furthermore, the local context will need to be taken into account while operationalizing linkages. As outlined in the Canada Health Act, public administration, comprehensiveness, universality, portability and accessibility must be kept in mind while operationalizing linkages [23]. A US study found that participants were more likely to complete an HIV self-test and get linked to care in the presence of a community health worker (e.g. respected peer in the community, familiar with cultural norms), compared to self-testing independently [24]. Various self-testing strategies are possible: supervised self-testing (set up self-testing kiosks in clinics or outreach community sites/vans), unsupervised self-testing (purchase of self tests from pharmacies or online sites, and provision of a toll free number for linkages to counselling and care) [2]. Respondents noted the importance of concrete percentages of (positive) self-testers not seeking counselling and care and failing to notify partners. Exploration of perspectives on self-testing from the target population to community-oriented implementation-research were deemed necessary to complement quantitative findings. Research on best models to self test is needed to bridge the “know-do gap” from research to policy and practice.

Another finding of the survey was that HIV self-testing was viewed by stakeholders as a challenge to the existing business models for HIV testing. It can be therefore be theorized, that since healthcare providers have long been charged with testing, diagnosis and treatment of HIV, they have a sense of ownership regarding the task of diagnosing HIV. They may also hold unique concerns towards this new approach based on their commitment to diagnosis and linkage. This theory may support findings from our ordinal logistic regression model, which compared the level of agreement on self-testing between frontline care providers and all other stakeholders. While frontline care providers were generally in favour of self-testing, all other stakeholders (e.g. AIDS community workers, researchers, etc.) were significantly more likely to respond in favour of self-testing. It is important to consider that screening strategies like self-testing are aimed to increase uptake of testing in the community as a triage test, and require confirmatory testing (similar to home pregnancy tests). Concerns regarding an overturn of the current model for HIV testing in Canada (by including self-testing) may be unjustified, as the role of self-testing is to provide an additional option to testing—not to replace current services.

Cost of self-testing was by stakeholders considered important for its uptake, consistent with other studies. In the US the high cost of oral HIV self-tests ($40–46 USD) impeded its widespread uptake, particularly due to high risk populations of low socioeconomic status [25]. This highlights of the concern that, despite readiness to test, high cost of self-test kits will present a barrier to their uptake. A majority of stakeholders expressed a preference for zero or low cost (<$20 CAD) HIV self-tests; findings concurred with another study [26]. This concern can be addressed by increasing availability of other self-tests (such as blood based self-tests), and competitive pricing.

Study limitations: While efforts were made to minimize selection bias, we were unable to calculate a response rate due to our convenience sampling methodology, and this study may have been at risk of non-response bias. Individuals who felt strongly about self-testing (either in favour or against) may have been more likely to respond to the survey than those who do not hold strong opinions. As this was an introductory/pilot survey, we additionally plan to expand upon the qualitative results by conducting semi-structured interviews among stakeholders, and a study on self-testing in Montreal. (CIHR operating Grant No MOP-123291).

## Conclusions

This study has highlighted the need to understand and address the local context (settings/environment/screening strategies) and constraints (legal, economic, political) when planning to implement innovative self-testing strategies. Understanding the role of self-testing as a screening strategy to improve awareness of HIV sero-status, increased engagement and access to care is extremely important; conventional testing strategies will always remain the mainstay to ensure patients receive treatment. Lastly, the integration of HIV self-testing within the existing HIV diagnostic environment in Canada will depend on cooperative engagement with all relevant stakeholders across all provinces.

In light of the recent WHO guidelines in favor of self-testing, it is now well established that the benefits of HIV self-testing extend beyond convenience, privacy, and empowerment, to increasing the knowledge of HIV serostatus [2, 20, 21, 27]. This aim is particularly relevant to Canada’s commitment to the UNAIDS 90-90-90 targets for ending the HIV/AIDS epidemic. This target aims for 90 percent of people infected with HIV being diagnosed, 90 percent linked to care and receiving treatment, and 90% with sustained viral suppression. We see the potential for HIV self-testing to become a tool to reach the 15–20% of undiagnosed Canadians, especially those who live in remote and isolated communities, and get them diagnosed and linked to care.
